# Production of red yeast rice rich in monacolin K by variable temperature solid fermentation of *Monascus purpureus*†

**DOI:** 10.1039/d3ra04374f

**Published:** 2023-09-12

**Authors:** Xinsong Yuan, Shan Gao, Yudie Tan, Jiyun Cao, Shiwei Yang, Bin Zheng

**Affiliations:** a School of Chemistry and Pharmaceutical Engineering, Hefei Normal University Hefei 230601 China yuanxs@zju.edu.cn

## Abstract

Hypercholesterolemia represents a serious public health problem as it significantly increases the risk of developing cardiovascular diseases. Monacolin K (MK) in red yeast rice is an active compound that can effectively lower plasma cholesterol. To enhance the yield of MK in solid state fermentation of *Monascus purpureus* HNU01, the effects of different variables were systematically examined in single-factor experiments. The optimal conditions for the production of red yeast rice rich in MK were as follows: initial pH value 5.5, initial moisture content 40% w/w, glucose 50 g L^−1^, peptone 20 g L^−1^, MgSO_4_ 0.5 g L^−1^, KH_2_PO_4_ 1 g L^−1^, variable temperature fermentation (30 °C for the first 3 days and then 24 °C for 15 days), total fermentation time of 18 days, and additional water added at day 4 at 10% w/w. Under the above optimized conditions, the MK content of red yeast rice produced by fermentation was 9.5 mg g^−1^. No citrinin was detected in any of the batches of fermentation products. The results will be useful for the large-scale production of high-quality red yeast rice with health benefits for consumers.

## Abstract 2

Hypercholesterolemia represents a serious public health problem as it significantly increases the risk of developing cardiovascular diseases.

## Graphical abstract

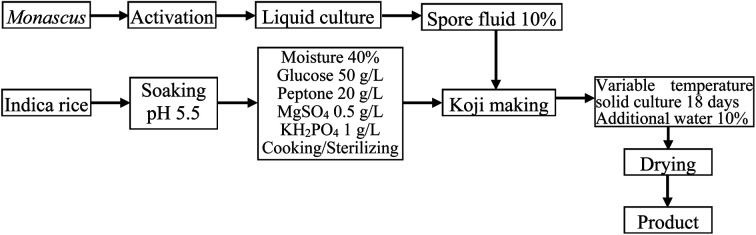

## 1 Introduction

In Asian countries, fermented products of the genus *Monascus* have been used as functional health foods for thousands of years.^1^ The most famous product is *Monascus*-fermented rice, also known as red yeast rice, red rice, red mold rice, ang-kak, anka, ankak, angkhak, angquac, and beni-koji.^2^ Red yeast rice contains many functional secondary metabolites, including pigments, monacolin, γ-aminobutyric acid, citrinin, and dimerumic acid.^1^ These metabolites have a diverse range of biological functions, including antimicrobial,^2^ anti-inflammatory,^3^ antiobesity,^4^ antidiabetes,^5^ and anticancer activities,^6^ and plasma cholesterol-^7,8^ and blood pressure-lowering^9^ effects.

Hypercholesterolemia is a primary risk factor for coronary artery disease, which is one of the leading causes of death in Western countries.^10,11^ Approximately 70% of the cholesterol in the human body is synthesized by the human body itself, so inhibiting excessive cholesterol synthesis in the body is an effective way to prevent cardiovascular disease.^12^ Monacolin K (MK, also known as lovastatin, mevinolin, and mevacor) in red yeast rice is an active compound that can effectively lower plasma cholesterol.^13^ It not only inhibits cholesterol biosynthesis, but also lowers blood cholesterol level in both humans and animals.^14^ Among the main metabolic pathways in which cholesterol is synthesized from acetyl-CoA, the rate-limiting step is the reduction of 3-hydroxy-3-methylglutaryl coenzyme A (HMG-CoA) to mevalonate. The enzyme that catalyzes this step is HMG-CoA reductase,^15^ and the amount of active enzyme directly affects the amount of cholesterol synthesized. Because the structure of MK is similar to that of the enzyme's substrate HMG-CoA, it is a potent competitive inhibitor of HMG-CoA reductase, the rate-limiting enzyme in cholesterol biosynthesis.^13^ Thus, MK can block the synthesis of mevalonate, thereby inhibiting cholesterol synthesis. It has been reported in the literature^16^ that the hexahydronaphthalene ring in the MK structure is an important group. Because of the existence of this group in MK, the enzyme has a stronger affinity for MK than for HMG-CoA, so MK can successfully block cholesterol synthesis and reduce the cholesterol level in plasma.^13,16^

The MK obtained from the fermentation of *Monascus* has two structural forms: an acid-form open-ring structure and a lactone-form closed-ring structure.^17,18^ Both acid MK and lactone MK can inhibit HMG-CoA reductase, but their effects are not exactly the same. Compared with lactone MK, acid MK has a stronger inhibitory effect on HMG-CoA reductase. Also, when lactone MK acts in the body, it requires the activity of a hydroxyesterase to hydrolyze it into the acid form. Long-term use of lactone-enriched MK products can increase the burden on the liver and kidneys, as well as causing vomiting, nausea, and other side effects.^19^ Therefore, the MK content and the proportion of acid MK out of total MK are important quality indicators for red yeast rice. More than a decade ago, the MK content in red yeast rice was generally not high, such as the 0.38 mg g^−1^ reported by Su *et al.*^1^ and the 0.53 mg g^−1^ reported by Wang *et al.*^14^ In recent years, the MK content in red yeast rice has significantly increased, such as the 16.3 mg g^−1^ reported by Lu *et al.*^19^ and the 12.9 mg g^−1^ reported by Lu *et al.*^20^ The Chinese Pharmacopoeia stipulated that the MK content in functional red yeast rice powders should not be less than 2.5 mg g^−1^.^21^ Compared with the Western medicine lovastatin (Lvs), the traditional Chinese medicine red yeast rice has fewer side effects, higher safety, and better comprehensive efficacy. Although the productivity of Lvs is as high as 1.27 g L^−1^ by *Aspergillus terreus*,^22^ the Lvs-containing broth produced by this microorganism is not generally used directly because *A. terreus* is not considered to be edible *per se*.^11^

Some *Monascus* species also produce citrinin, a nephrotoxic mycotoxin.^23^ Citrinin can cause enlargement of the kidneys and mitochondria, leading to widespread cell death.^24^ Therefore, a sufficiently low or undetectable citrinin content is a basic requirement for high-quality red yeast rice.

**Fig. 1:**
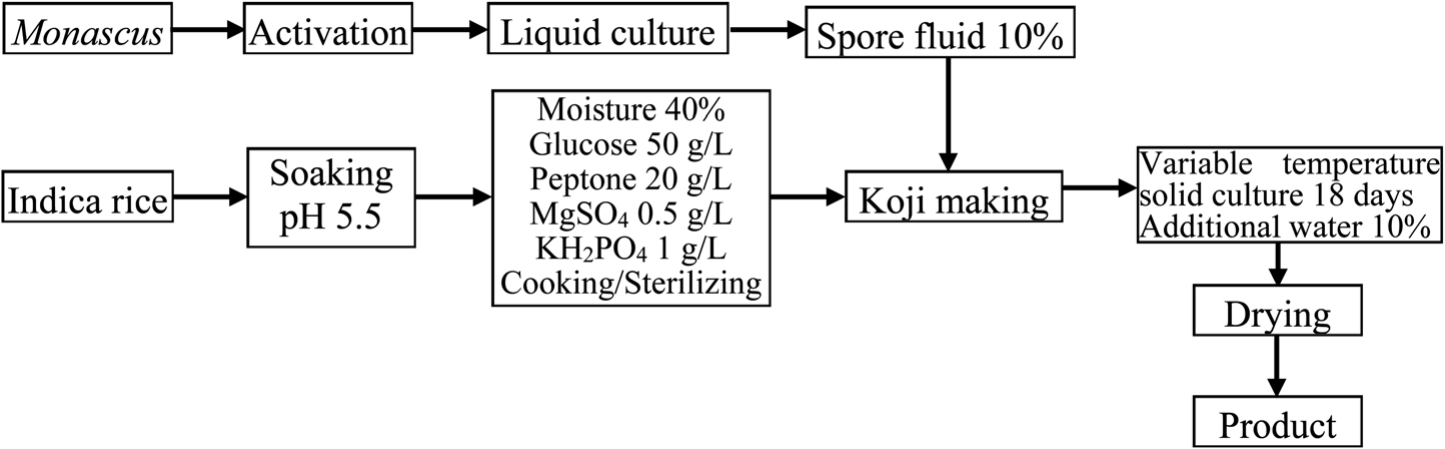
Production process of solid-state fermentation of red yeast rice.

Although liquid fermentation has many advantages such as a high degree of automation, low labor requirements, and a large production scale, the yield of MK obtained by submerged liquid fermentation is much lower than that of solid-state fermentation.^19,25^ Therefore, the aim of this work was to improve the solid fermentation process for producing high-quality red yeast rice. To this end, the effects of various factors including initial moisture content, initial pH value, fermentation period, medium composition, and solid culture temperature on the MK content were determined in single-factor experiments. The production process diagram is shown in Fig. 1.

## 2 Experimental

All reagents were purchased from the Sinopharm Chemical Reagent Co., Ltd (Shanghai, China) unless otherwise stated. Red yeast rice production was carried out using strain *M. purpureus* HNU01, which was preserved in our laboratory.

### 2.1 Seed cultures

Seed cultures were prepared by transferring a whole loopful of spores from a PDA agar (2% w/w) slant into a 500 mL Erlenmeyer flask containing 100 mL basal medium (60 g glucose, 25 g peptone, 2 g NaNO_3_, 1 g KH_2_PO_4_, 1 g MgSO_4_, 1 g CaCl_2_ in 1 L distilled water, pH adjusted to 6.0, steam sterilized at 115 °C for 30 min). The flask cultures were incubated at 30 °C for 72 h with shaking at 110 rpm.

### 2.2 Soaking, cooking, and sterilization of rice

Indica rice was purchased from a local supermarket and was used as the substrate for *Monascus*-fermented rice production under solid-state conditions. A 50 g sample of rice was added to a 250 mL Erlenmeyer flask. The rice was soaked in lactic acid aqueous solution for 12 h, then drained and dried with gauze. The effects of the initial pH value (4.0, 4.5, 5.0, 5.5, 6.0, 6.5) and initial moisture content (35%, 40%, 45%, 50%, 55%, 60% w/w, based on dry rice) on the MK content in the red yeast rice product were investigated.

Spore liquid was filtered from the seed culture medium with sterilized absorbent cotton and then added to dry rice at a rate of 10% (w/w) of the dry rice weight. The optimized culture medium had the following composition (g per L): glucose 50, peptone 20, MgSO_4_ 0.5, KH_2_PO_4_ 1. The culture medium was steam-sterilized at 115 °C for 30 min and then cooled before use. The effects of the concentrations (g L^−1^) of glucose (20, 30, 40, 50, 60, 70), peptone (10, 15, 20, 25, 30, 35), MgSO_4_ (0, 0.25, 0.5, 0.75, 1.0, 1.25), and KH_2_PO_4_ (0, 0.5, 1.0, 1.5, 2.0, 2.5) in the culture medium on MK content were investigated.

The flask mouth was wrapped with eight layers of gauze, and the rice was steam-sterilized at 115 °C for 30 min.

### 2.3 Solid-state fermentation for MK production

A *Monascus* ball was stirred and dispersed in culture medium with a glass rod, and the mixture was filtered through sterilized cotton wool to obtain a monodisperse spore suspension. After cooling, the sterile rice substrate was inoculated with 10% spore suspension and the mixture was stirred well with a glass rod. Then, the inoculated substrate was cultivated in a biological incubator at suitable temperature for a period of time. Fermentation was carried out at 30 °C for 3 days, then at 24 °C for an additional 15 days, with mixing every 2 days. A suitable amount of sterile water was added to the culture medium on the fourth day. The effects of fermentation temperature (constant or variable temperature), fermentation time (days: 15, 16, 17, 18, 19, 20), and amount of water added (2.5%, 5.0%, 7.5%, 10.0%, 12.5%, 15.0% w/w) on MK content were investigated.

After fermentation, the MK and citrinin contents in the red yeast rice product were measured.

### 2.4 MK and citrinin content analysis

#### 2.4.1 Determination of MK concentration

The lactone-form MK standard solution was prepared by diluting 5.0 mg standard MK (lovastatin, B25912, 1 mg, Shanghai McLean Biochemical Technology Co., Ltd, China) in 25 mL methanol to obtain a 200 mg L^−1^ lactone MK stock solution. The stock solution was diluted to obtain standard solutions with concentrations of 40 mg L^−1^, 20 mg L^−1^, 4 mg L^−1^, 2 mg L^−1^, 0.2 mg L^−1^, and other concentrations.

The acid-form MK standard solution was prepared according to Kysilka and Ken,^26^ as follows: 5.0 mg of the lactone MK standard product described above was dissolved in 4 mL methanol, then 20 mL 0.2 mol L^−1^ NaOH solution was added and the mixture was sonicated for 30 min. The pH was adjusted to 6.0 with 3 mol L^−1^ phosphoric acid, and the volume was adjusted to 25 mL with methanol to obtain a 200 mg L^−1^ acid MK stock solution. After dilution, standard solutions of 40 mg L^−1^, 20 mg L^−1^, 4 mg L^−1^, 2 mg L^−1^, 0.2 mg L^−1^ were obtained.

The MK standard solutions with the concentrations described above were analyzed by high-performance liquid chromatography using an LC-20A HPLC instrument (Shimadzu, Kyoto, Japan). The retention time and peak area of each product were recorded. A corresponding standard curve was prepared for analyses of the MK content in red yeast rice samples.

The methods for determination of the MK concentration in red yeast rice fermented under different conditions were as described by Zhang *et al.*^27^ The red yeast rice samples were oven-dried at 50 °C for 12 h, and then ground into powder and passed through a 100-mesh sieve. A 0.50 g portion of the powder was weighed into a stoppered flask, and 10 mL 75% v/v ethanol was added as the extractant. Extraction was performed for 1 h at 55 °C with shaking at 120 rpm. After centrifugation at 4000 rpm for 10 min, the supernatant was collected and made up to 10 mL with extractant. The extract was filtered through 0.22 μm membrane and then analyzed by HPLC. The HPLC conditions were as follows: Shimadzu Shim-pack GIST C_18_ column (4.6 × 250 mm, 5 μm, column temperature 28 °C); mobile phase of methanol–water (385 : 115, v/v, pH adjusted to 2.5 with H_3_PO_4_); flow rate, 1 mL min^−1^; column temperature, 28 °C; injection volume, 10 μL; detection using a SPD-20A UV detector (Shimadzu) at 237 nm. The MK content was calculated as follows: MK content *t* (mg g^−1^) = 0.05*c* (mg L^−1^).

Where MK content *t* (mg g^−1^) is the total mass of MK (acid form and lactone form) per gram of dry red yeast rice, and *c* is the MK concentration in the extract (mg L^−1^).

#### 2.4.2 Determination of citrinin concentration

The citrinin concentration was determined as described by Xu *et al.*^28^ The citrinin standard solution was prepared using a citrinin standard sample (B25912), which was purchased from Shanghai Yuanye Biotechnology Co., Ltd, China. A 1.0 mg portion of citrinin standard product was dissolved in acetonitrile and then diluted to 10 mL to obtain a 100 mg L^−1^ stock solution. The stock solution was diluted to obtain solutions with concentrations of 20 mg L^−1^, 10 mg L^−1^, 2 mg L^−1^, 1 mg L^−1^, 0.2 mg L^−1^. Each solution was analyzed by HPLC equipped with a fluorescence detector. The retention time and peak area were obtained for each sample, and the concentration and peak area were used to construct a citrinin standard curve.

To detect citrinin, dry red yeast rice was ground into a powder, sieved through a 100-mesh sieve, and 0.50 g of the powder was accurately weighed into a stoppered flask. Then, 8 mL extractant (toluene–ethyl acetate–formic acid 7 : 3 : 1) was added to the flask, and the mixture was sonicated for 5 min. The mixture was then centrifuged at 4000 rpm for 5 min, and the supernatant was transferred into a 25 mL volumetric flask. This extraction step was repeated twice more and the supernatants were combined. The supernatants were mixed and adjusted to a final volume of 25 mL. The supernatant was filtered through a 0.22 μm membrane, and the citrinin content was determined by HPLC.

The conditions for HPLC analysis of citrinin were as follows: Shimadzu Shim-pack GIST C_18_ column (4.6 × 250 mm, 5 μm, column temperature 28 °C); mobile phase of acetonitrile–water (45 : 55, v/v, pH was adjusted to 2.5 with H_3_PO_4_); flow rate, 1 mL min^−1^; injection volume, 10 μL; detection with a RF-20A fluorescence detector (Shimadzu) with excitation and emission wavelengths of 331 nm and 500 nm, respectively.

## 3 Results

### 3.1 Influence of various factors on total MK content in red yeast rice

With the MK content in red yeast rice as the main index, the influence of various factors on total MK (acid form and lactone form) content was investigated in single-factor experiments. The factors included initial pH value, initial moisture content, the concentrations of glucose, peptone, MgSO_4_, and KH_2_PO_4_ in the medium, the fermentation temperature and time, as well as the amount of additional water added during fermentation.

The effects of the initial pH value and initial moisture content on MK content in red yeast rice are presented in Fig. S1a and b.† The highest MK content in red yeast rice was obtained with an initial pH value of 5.5 and an initial moisture content of 40% (w/w).

The composition of the culture medium also significantly affected the MK content. The highest MK content in red yeast rice was obtained with the following concentrations of media components: 50 g L^−1^ glucose (Fig. S1c†), 20 g L^−1^ peptone (Fig. S1d†), 0.5 g L^−1^ MgSO_4_ (Fig. S1e†), and 1.0 g L^−1^ KH_2_PO_4_ (Fig. S1f†).

Changing the fermentation temperature from high to low is beneficial for increasing the production of secondary metabolites. Of the six types of temperature conditions, type 1 (30 °C for the first 3 days and then 24 °C) was most beneficial for increasing the MK content of red yeast rice (Fig. S1g†). Compared with type 1, type 2 (32 °C for the first 3 days and then 24 °C) resulted in a slightly lower MK concentration. The highest MK content was obtained at 18 days of fermentation, and prolonging the fermentation time resulted in almost no change in MK content (Fig. S1h†). Additional water was added on day 4 of the fermentation period, and the amount added significantly affected the MK content in red yeast rice. The highest MK content was obtained by supplying additional water at 10% w/w. Increasing the amount of additional water resulted in lower concentrations of MK in the red yeast rice (Fig. S1I†).

The results of the single-factor experiments indicated that the optimal conditions for production of red yeast rice rich in MK were as follows: initial pH value 5.5, initial moisture content 40% (w/w), medium containing glucose 50 g L^−1^, peptone 20 g L^−1^, MgSO_4_ 0.5 g L^−1^, and KH_2_PO_4_ 1 g L^−1^, variable temperature fermentation (30 °C for the first 3 days and then 24 °C), fermentation time of 18 days, and 10% w/w additional water added on day 4. Under these optimized conditions, the MK content in fermented red yeast rice was 9.5 mg g^−1^.

### 3.2 Citrinin in red yeast rice products

In this study, we used the HPLC chromatogram of a standard substance for citrinin with a concentration of 0.2 mg L^−1^ as a reference, and no citrinin was detected in the seven single factor experimental fermentation products.

### 3.3 Proportion of acid MK out of total MK in red yeast rice products

In this study, the proportion of acidic MK out of total MK ranged from 65% to 90%. The concentration of MgSO_4_ in the culture medium significantly affected the proportion of acidic MK (Fig. S2†). The highest proportion of acidic MK (90%) was obtained with 0.5 g L^−1^ MgSO_4_ in the medium.

## 4 Discussion

The MK content in the solid-state fermentation product is more than 20 times higher than that in the liquid fermentation product.^29^ Soft rice, including glutinous rice, has a high viscosity and is prone to clumping, which is not conducive to the growth of *Monascus*. Indica rice has good breathability and water absorption, and can maintain appropriate humidity. Therefore, in this work, we used Indica rice as the substrate to produce red yeast rice rich in MK by solid-state fermentation.

The initial pH affects the solid-state fermentation of MK. *Monascus* grows best in acidic culture conditions. It has been reported that soaking rice in lactic acid solution can effectively reduce bacterial contamination during the fermentation process.^30^ Moreover, changes in pH can affect enzyme activity, thereby affecting the rate of substrate utilization and cell structure.^31^ Our experimental results indicate that red yeast rice produces the highest concentrations of MK under weak acidic conditions, while pH values that are too high or too low are not suitable.

Our results also show that the initial moisture content also significantly affects the MK content. The highest MK content in red yeast rice was obtained with an initial moisture content of 40% w/w. When the initial moisture content was below 40% w/w, the culture medium became dry and hard, which inhibited bacterial growth and resulted in low MK production in the fermentation product. When the initial moisture content was too high, viscous substances appeared at the bottom of the culture medium and MK production was inhibited.^32^

Adding certain additional nutrients to the rice substrate can significantly increase the yield of MK. Glucose and peptone are commonly used carbon/nitrogen sources for cultured microorganisms. The glucose and peptone content significantly affected the yield of MK, and concentrations that were too high or too low were not conducive to MK production.^33^ If the carbon supply is limited, the strain cannot obtain sufficient energy, and its growth and reproduction are restricted, thereby reducing MK production. If the carbon supply is excessive, the strain is always in a good growth environment, but the conditions are not conducive to MK production. Phosphorus is an important component of nucleic acids, phosphate, and coenzymes.^34^ It is also a commonly used nutrient in fermentation media. In this study, the amount of phosphate added affected MK production. Excessive phosphate led to a decrease in yield, and was not conducive to MK accumulation.

Mg^2+^, as an activator of microbial enzymes, participates in many important metabolic processes, including carbohydrate metabolism, nucleic acid synthesis, and phosphate conversion. It is also involved in the regulation of cell cycle, cell proliferation, and differentiation. Our results show that the Mg^2^ concentration significantly affected the growth of *Monascus*, its ability to produce metabolites, and the proportion of acid MK. The proportion of acidic MK is related to both the strain itself and the fermentation conditions. In this study, the proportion of acidic MK produced by HNU01 ranged from 65% to 90%, significantly higher than the proportion in many red yeast rice products. It is of great significance to develop a red yeast rice production process in which the proportion of acid MK can be controlled to meet the needs of various users. To our knowledge, there have been no reports on fermentation methods for red yeast rice in which the proportion of acid MK is controlled. The formation of acid MK is related to the activity of polyketases and the concentration of cyclic adenosine monophosphate.^35^ Thus, we speculate that Mg^2+^ may participate as a co-factor for enzymes involved in the synthesis of acid MK. However, the specific mechanism by which Mg^2+^ affects the acid MK ratio is still unclear, and warrants further investigation.

A variable temperature fermentation strategy with a high temperature in the early stage and a low temperature in the late stage is conducive to improving the yield of secondary metabolites. A relatively high temperature (30 °C) is beneficial for yeast growth, but unfavorable for enzyme activity. *Monascus* may grow faster at slightly higher temperatures (35 or 37 °C), but temperatures above 30 °C may have adverse effects on protein folding, thereby negatively affecting enzyme activity.^36^ Thus, the temperature was 30 °C for the first 3 days, and then decreased to 24 °C for the final 15 days of fermentation. With prolonged fermentation time, the MK content gradually increased, and it reached the maximum at 18 days of fermentation. Further extension of the fermentation time resulted in a slight decrease in MK content. In the later stage of fermentation, the fermentation substrate was depleted, and yeast cell growth was inhibited. This led to the consumption of fermentation products, including MK, resulting in a decrease in MK content. Maintaining an appropriate amount of moisture in the substrate is beneficial for yeast cell growth and secondary metabolite production. As fermentation progresses, due to the consumption of bacterial growth and the evaporation of water, appropriate replenishment of water is necessary. Proper moisture content can maintain a soft and fluffy substrate, which is conducive to bacterial respiration and material exchange, thereby increasing MK production. In this study, providing additional water (10% w/w) on day 4 was shown to significantly improve the MK content.

There are two main sources of toxins like citrinin, one is that the strain carries the gene of the synthesis of these substances, and the other is the pollution of the production process. If no other microorganisms are present, then the production (or content) of citrinin in red yeast rice is mainly determined by *Monascus* itself. For this HNU01 strain, as no citrinin was detected in any of the batches of fermentation products under our HPLC analysis conditions, we speculate that this strain does not carries the gene, or this gene is silenced under current fermentation conditions.

As a technology that has been passed down for thousands of years in East Asia, the production of red yeast rice rich in MK by solid-state fermentation should be possible on a large scale. Compared with literature reports, the MK content in this work is not the highest. This is not only related to the fermentation process, but may also be related to the strain itself. However, the red yeast rice produced in this work does not contain citrinin, has a high MK content, and a high proportion of acidic MK. The main purpose of this study was to explore the key factors affecting MK content in red yeast rice obtained using a solid fermentation process, and to determine the mechanisms by which individual factors affect the MK content based on information in the literature and experience.

## 5 Conclusions

The conditions for the production of red yeast rice rich in MK were optimized as follows: initial pH value 5.5, initial moisture content 40% w/w, glucose 50 g L^−1^, peptone 20 g L^−1^, MgSO_4_ 0.5 g L^−1^, KH_2_PO_4_ 1 g L^−1^, variable temperature fermentation (30 °C for the first 3 days and then 24 °C for 15 days), total fermentation time of 18 days, and additional water added at day 4 at 10% w/w. Under the above conditions, the MK content of red yeast rice produced by fermentation was 9.5 mg g^−1^. The concentration of MgSO_4_ in the culture medium significantly affected the proportion of acidic MK. The highest proportion of acidic MK (90%) was obtained with 0.5 g L^−1^ MgSO_4_ in the medium. No citrinin was detected in any of the batches of fermentation products.

## Conflicts of interest

There are no conflicts to declare.

## Supplementary Material

RA-013-D3RA04374F-s001
