# Supplementary material for: Production of red yeast rice rich in monacolin K by variable temperature solid fermentation of Monascus purpureus
Source: RSC Adv. 2023 Sep 12;13(39):27303–8. doi: 10.1039/d3ra04374f (PMC10496031; doi:10.1039/d3ra04374f)
Supplement: RA-013-D3RA04374F-s001 [file RA-013-D3RA04374F-s001.pdf]

Electronic Supplementary Material (ESI) for RSC Advances.
This journal is © The Royal Society of Chemistry 2023

Supplemental materials

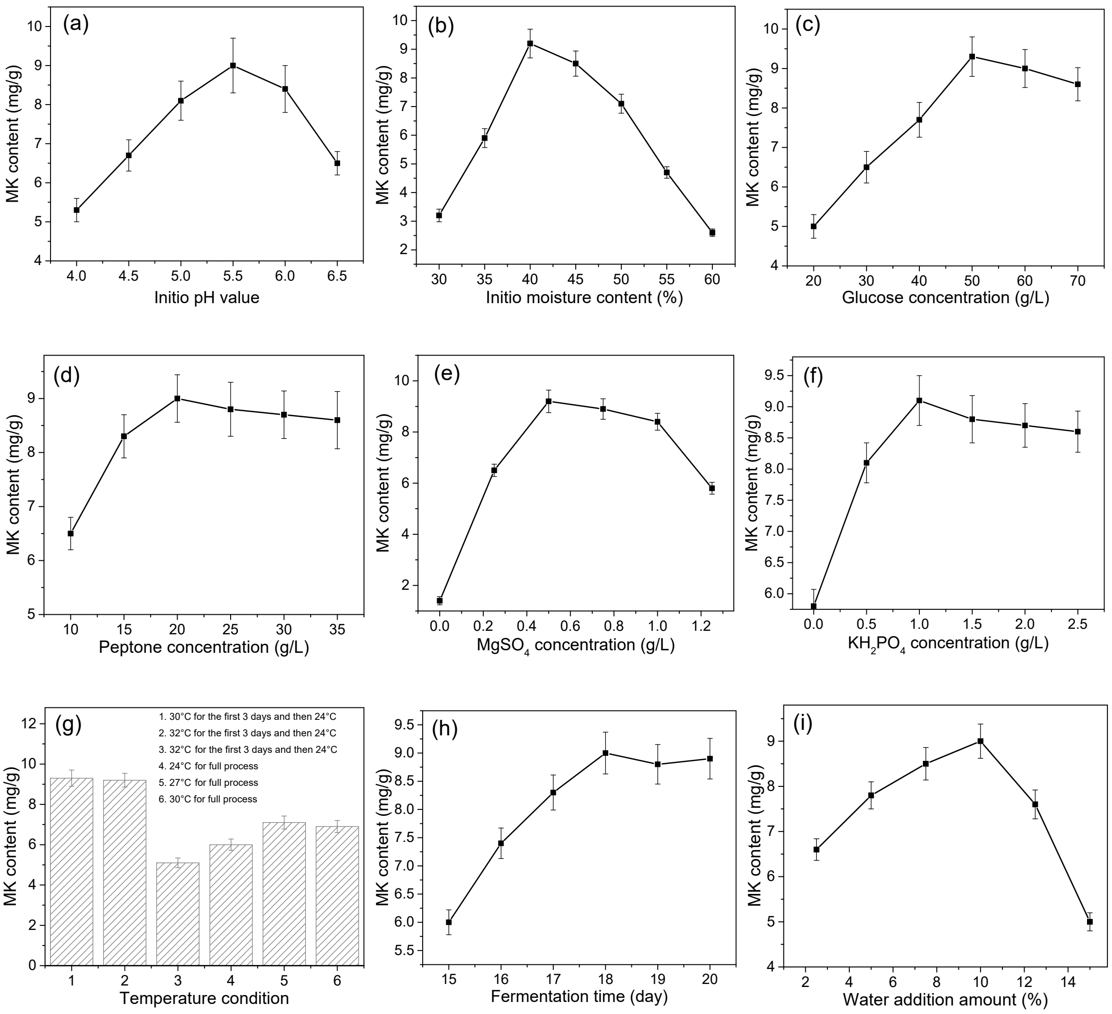

Figure S1. Influence of various factors on MK content in red yeast rice: initial pH value (a), initial moisture content (b), glucose (c), peptone (d), $MgSO_4$ (e) and KH_2PO_4 (f) concentration, fermentation temperature (g), fermentation time (h) and amount of additional water added (i)

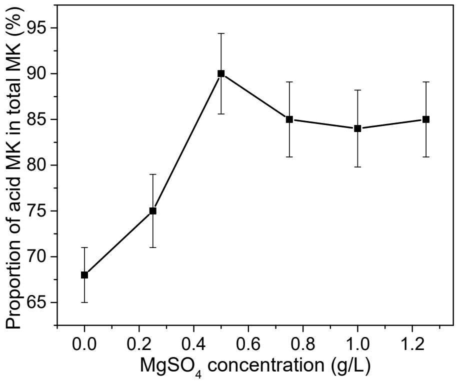

Figure S2. Effect of $MgSO_4$ concentration on the proportion of acid MK out of total MK in red yeast rice

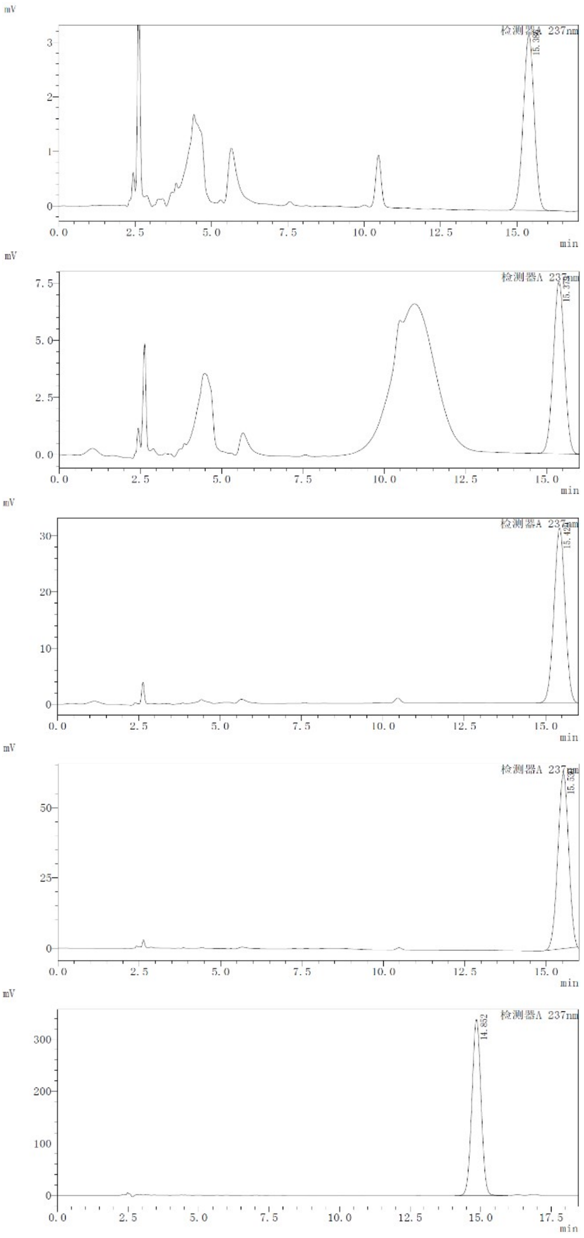

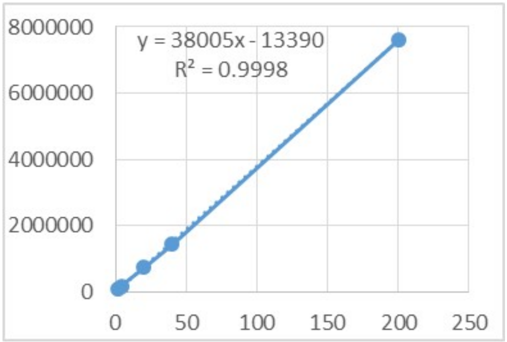

Concentration (mg/L)	Area	Hight
2	80161	3213
4	180671	7544
20	740406	31152
40	1440681	62201
200	7600410	338672

Figure S3. HPLC chromatograms and standard curves of acidic MK standard substance

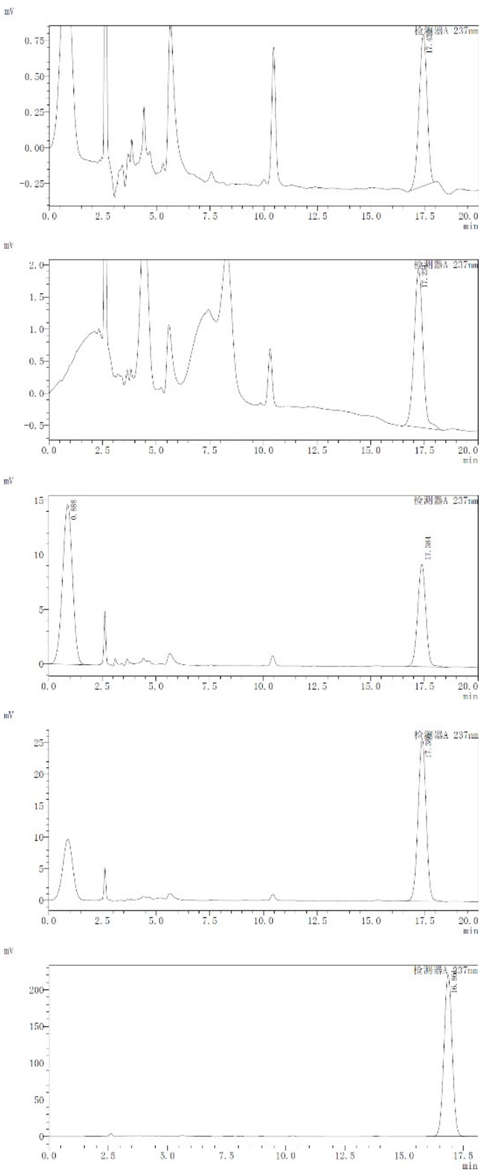

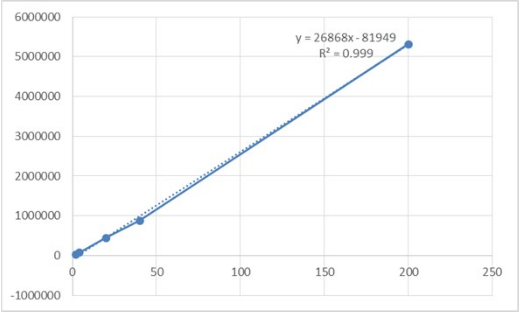

Concentration (mg/L)	Area	Hight
2	27674	1057
4	70723	2470
20	448205	9384
40	876379	25776
200	5314285	338672

Figure S4. HPLC chromatograms and standard curves of lactone MK standard substance

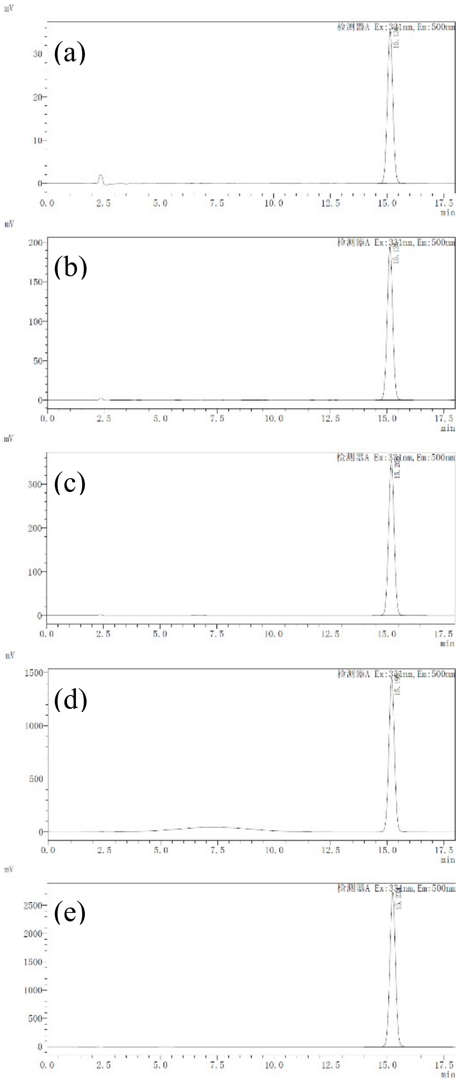

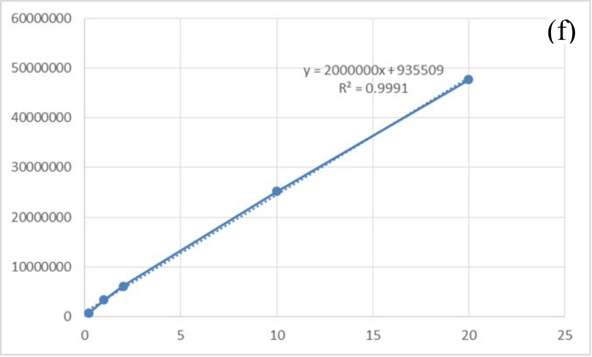

Concentration (mg/L)	Area	Hight
0. 2	604522	35396
1	3342460	195643
2	6114654	353070
10	25177247	1454104
20	47722768	2737368

Figure S6. HPLC chromatograms (a: 0.2 mg/L, b: 1 mg/L, c: 2 mg/L, d: 10 mg/L, e: 20 mg/L) and standard curves (f) of citrinin standard substance

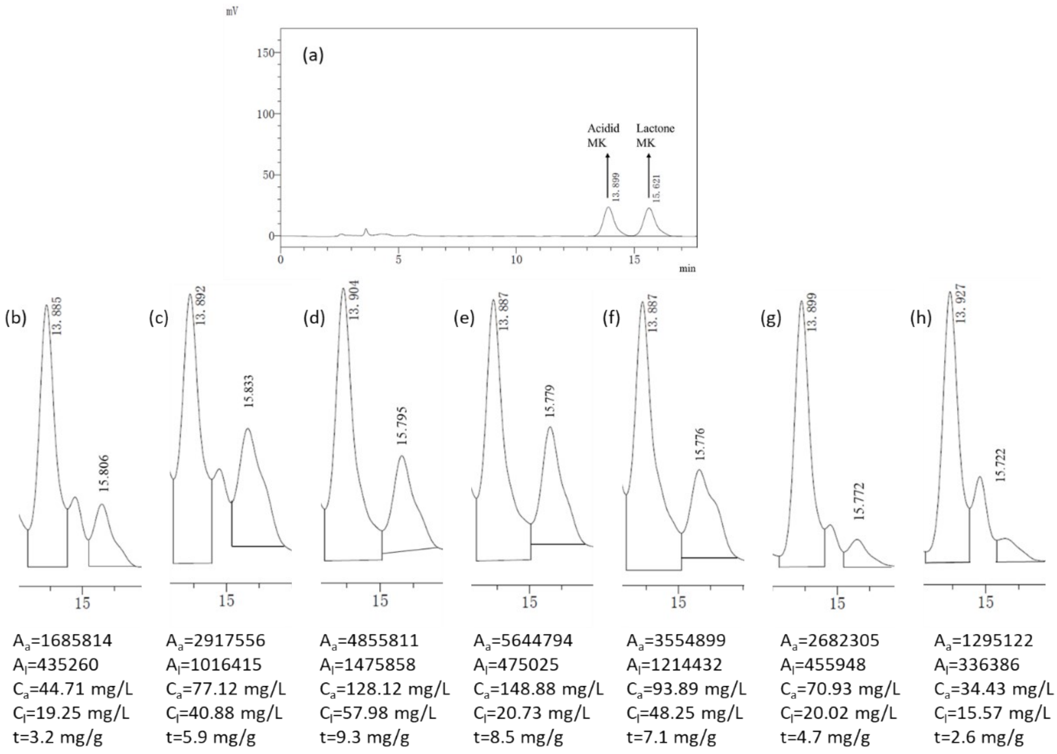

Figure S5. HPLC chromatograms of MK standard substance (a, Acid and Lactone type), and fermentation products MK for different initial moisture content (%): 30 (b), 35 (c), 40 (d), 45 (e), 50 (f), 55 (g) and 60 (h). Where A is peak area, t (mg/g) is the total mass of MK (acid form and lactone form) per gram of dry red yeast rice, and *c* is the MK concentration in the extract (mg/L).

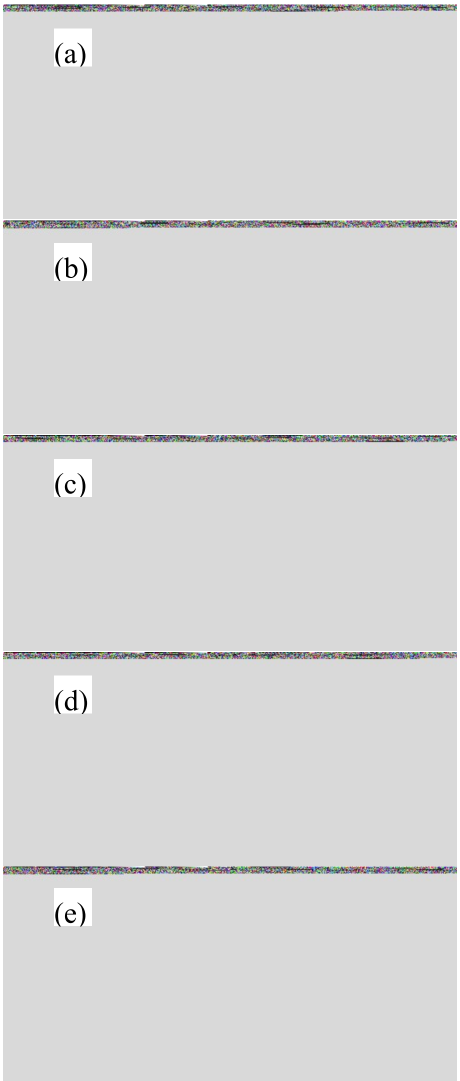

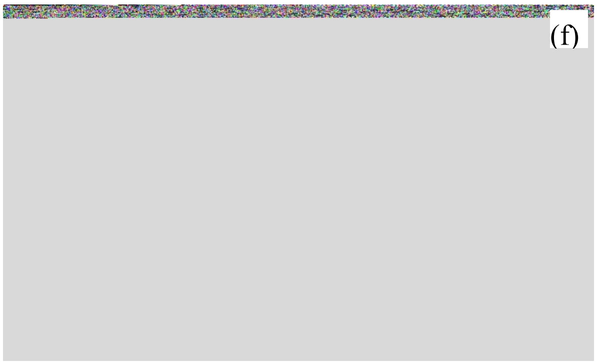

Concentration (mg/L)	Area	Hight
0.2	604522	35396
1	3342460	195643
2	6114654	353070
10	25177247	1454104
20	47722768	2737368

Figure S6. HPLC chromatograms (a: 0.2 mg/L, b: 1 mg/L, c: 2 mg/L, d: 10 mg/L, e: 20 mg/L) and standard curves (f) of citrinin standard substance

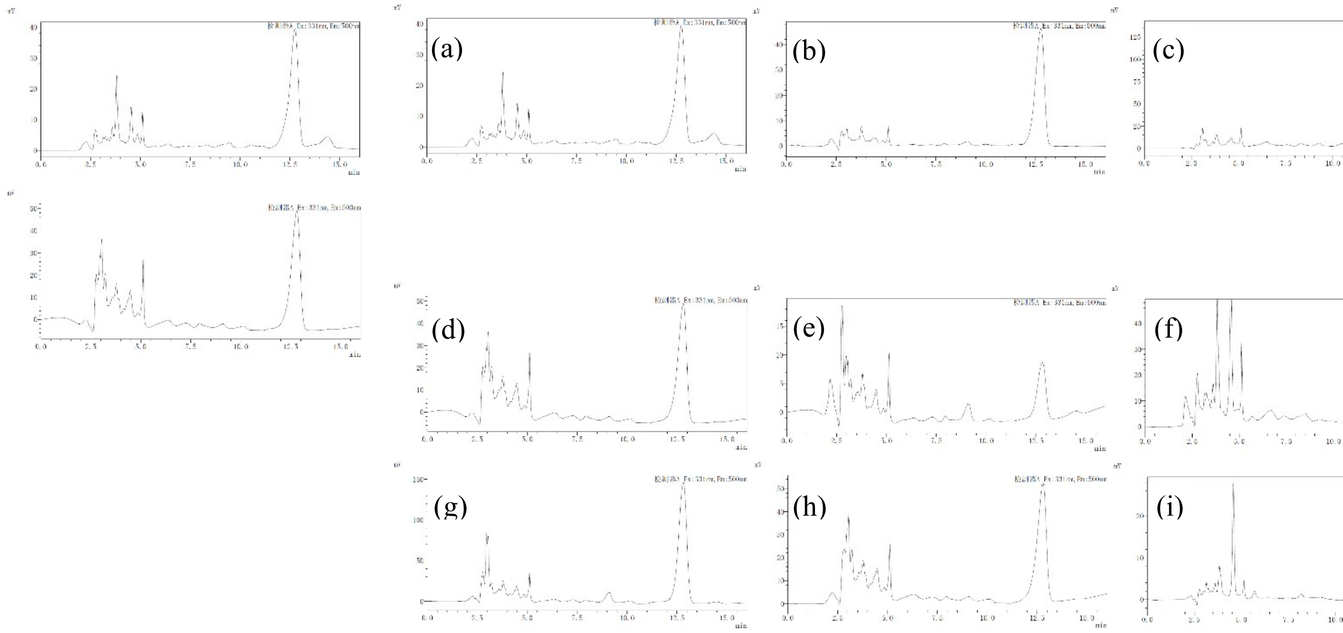

Figure S7. HPLC chromatograms of citrinin in products under various single factor experimental optimization conditions: initial pH value 5.5 (a), initial moisture content 40% w/w (b), glucose 50 g/L (c), peptone 20 g/L (d), $MgSO_4$ 0.5 g/L (e), KH_2PO_4 1 g/L (f), variable temperature fermentation (30 °C for the first 3 days and then 24 °C for 15 days, g) , total fermentation time of 18 days (h), and additional water added at day 4 at 10% w/w (i)
